# Changes in Plasma Phospholipid Metabolism Are Associated with Clinical Manifestations of Systemic Sclerosis

**DOI:** 10.3390/diagnostics11112116

**Published:** 2021-11-15

**Authors:** Marija Geroldinger-Simić, Thomas Bögl, Markus Himmelsbach, Norbert Sepp, Wolfgang Buchberger

**Affiliations:** 1 Department of Dermatology, Ordensklinikum Linz Elisabethinen, 4020 Linz, Austria; norbert.sepp@ordensklinikum.at; 2 Faculty of Medicine, Johannes Kepler University Linz, 4040 Linz, Austria; 3 Institute for Analytical and General Chemistry, Johannes Kepler University Linz, 4040 Linz, Austria; thomas.boegl@jku.at (T.B.); markus.himmelsbach@jku.at (M.H.); wolfgang.buchberger@jku.at (W.B.)

**Keywords:** systemic sclerosis, lipidomics, skin fibrosis, lung fibrosis, calcinosis cutis, digital ulcers, sicca, plasmalogens, sphingomyelin, phospholipids

## Abstract

Systemic sclerosis (SSc) is an autoimmune disease with fibrosis of the skin and/or internal organs, causing a decrease in quality of life and survival. There is no causative therapy, and the pathophysiology of the SSc remains unclear. Studies showed that lipid metabolism was relevant for autoimmune diseases, but little is known about the role of lipids in SSc. In the present study, we sought to explore the phospholipid profile of SSc by using the lipidomics approach. We also aimed to analyze lipidomics results for different clinical manifestations of SSc. Experiments were performed using high-performance liquid chromatography coupled to mass spectrometry for the lipidomic profiling of plasma samples from patients with SSc. Our study showed, for the first time, significant changes in the level of phospholipids such as plasmalogens and sphingomyelins from the plasma of SSc patients as compared to controls. Phosphatidylcholine plasmalogens species and sphingomyelins were significantly increased in SSc patients as compared to controls. Our results also demonstrated a significant association of changes in the metabolism of phospholipids (phosphatidylcholine and phosphatidylethanolamine plasmalogens species and sphingomyelins) with different clinical manifestations of SSc. Further lipidomic studies might lead to the detection of lipids as new biomarkers or therapeutic targets of SSc.

## 1. Introduction

Systemic sclerosis (SSc) is a chronic autoimmune disease that leads to fibrosis of the skin and/or internal organs and endothelial damage [1]. The most frequent cause of death in SSc patients is progressive lung fibrosis (LF). The quality of life in SSc patients is decreased due to multi-organ manifestations of SSc, such as thickening of the skin, chronic digital ulcers (DU), pain and superinfections due to calcinosis cutis (CC), dyspnea due to lung disease or dry mouth, and eyes due to sicca mucositis [2]. Based on the skin involvement, there are two major forms of SSc, limited cutaneous SSc (lcSSc), where skin fibrosis is limited to the distal extremities and face, and diffuse cutaneous SSc (dcSSc) with generalized skin fibrosis [2]. Clinical manifestations and autoantibody profiles are often specific for these two subsets of SSc. Anti-centromere antibodies (ACA) are frequently associated with lcSSc and anti-Scl70-antibodies (anti-topoisomerase I-antibodies) with dcSSc. Although management of SSc has improved during the last years [3,4], there is no causative treatment for SSc, and the pathophysiology is still unclear.

There is emerging evidence on the relevance of lipids in autoimmune and inflammatory diseases. In systemic lupus erythematosus (SLE), alteration of serum lipids, in particular lipid plasmalogens, were reported [5]. Lipidomic studies showed an altered phospholipid metabolism in sera from patients with multiple sclerosis compared to controls and suggested the crucial role of lipids in the pathophysiology and severity of multiple sclerosis [6,7,8]. Lipids from serum or plasma, such as phospholipids and lysophospholipids, were different in chronic diseases such as rheumatoid arthritis (RA) or psoriasis, as compared to controls. It was also possible to differ between RA and Lyme arthritis based on the lipidomics profile [9]. Lipid profiles were also altered in patients with polymyositis and dermatomyositis as compared to controls [10].

In the literature, there are only limited data on the role of lipids in SSc. A study showed low HDL levels in patients with lcSSc with positive ACA [11]. Moreover, significantly higher serum levels of phospholipids like arachidonoyl (20:4)—Lysophosphatidic acid (LPA), and sphingosine 1-phosphate (S1P) in SSc versus controls were reported [12]. In LPA1 knockout mice, skin fibrosis was reduced in the bleomycin-induced scleroderma model [13,14,15]. Moreover, SSc patients’ altered fatty acid profile in plasma and dendritic cells was reported [16].

Phospholipids are essential for building membranes of cells and organelles. Numerous functions of phospholipids on the cellular level, such as regulation of cell shape, cell migration, and intercellular communication, are well documented [17]. Signaling pathways of phospholipids are very complex, and small changes in phospholipid levels can strongly influence cell survival [17,18]. Studies showed the involvement of phospholipids in cardiovascular and metabolic diseases, cancer, inflammatory and autoimmune disorders, and neurodegenerative diseases [8,17,19,20].

Subgroups of phospholipids such as plasmalogens and sphingomyelins (SM) play an important role in vital organs. Plasmalogens represent a significant part of membranes in neurons, blood cells, and muscles (including the heart) and are responsible for storing inflammatory mediators and regulating oxidative stress [21,22,23]. Defects in the synthesis of plasmalogens lead to damages in the brain, lung, kidney, and heart [20]. The most abundant phospholipids in humans are phosphatidylcholine (PC), with 40–50% of total phospholipids, followed by phosphatidylethanolamine (PE), which is particularly frequent in mitochondrial membranes [17]. Both PC and PE species with one acyl and one alkenyl side chain (called PC ae and PE ae) are known as phospholipids plasmalogens. Sphingolipids are membrane structural components and are involved in cardiovascular and metabolic diseases [24,25]. Accumulation of SM due to genetic defects leads to multi-organ damage in the brain, lung, and liver [26].

Our aim in this study was to explore the phospholipid profile in the plasma of patients with SSc using the lipidomics approach. Furthermore, this study aimed to analyze changes in phospholipid metabolism in different clinical manifestations of SSc.

## 2. Materials and Methods

### 2.1. Study Subjects and Plasma Samples

Patients with systemic sclerosis and control subjects without systemic sclerosis were recruited at the Department of Dermatology, Ordensklinikum Linz Elisabethinen, Linz, Austria. Patients were assessed during routine annual checkups (laboratory, serological tests, and assessment of organ involvement). Diagnosis of SSc was performed following the 2013 classification criteria for SSc by the American College of Rheumatology (ACR) and the European League Against Rheumatism (EULAR) [2]. Informed consent was obtained from all subjects involved in the study. This project was approved by the Ethics Committee of the Johannes Kepler University Linz, Austria.

The modified Rodnan skin score (mRSS) was used for the measurement of skin fibrosis [27]. Lung fibrosis (LF) was assessed using high-resolution computed tomography (HRCT) scans and pulmonary function tests. Calcinosis cutis (CC) was evaluated clinically and using an X-ray. Plasma samples were stored at 4 °C for a maximum of four hours and then at −80 °C before further processing.

### 2.2. Sample Preparation

Lipids were extracted by a modified Folch protocol [28]. A total of 75 μL plasma was mixed with 1.5 mL chloroform/methanol (2:1) and shaken for 10 min at 4 °C. Subsequently, 300 μL 5% acetic acid was added for phase separation, and the sample was shaken for 30 min and centrifuged (4200× *g*, 10 min, 4 °C). The lower layer was transferred into a 2 mL safe lock-tube, while the upper phase was mixed with 500 μL chloroform and shaken for 30 min. Again, after centrifugation (4200× *g*, 10 min, 4 °C), the lower phase was collected and combined with the solution in the safe-lock tube. The organic phases were dried under a gentle nitrogen stream, reconstituted in 150 μL methanol/chloroform (9:1), and stored at −80 °C until analysis.

### 2.3. HPLC-IM-QTOF Measurement

We explored the phospholipid profile in the plasma of patients with SSc using a lipidomics approach utilizing a high-performance liquid chromatography coupled to an ion mobility quadrupole time-of-flight (HPLC-IM-QTOF). The HPLC utilized an Eclipse Plus C8 column (3.5 μm, 3.0 mm × 150 mm, Agilent, Waldbronn, Germany) equipped with a C18 guard column (4 × 3.0 mm, Phenomenex). For measurement, a 1290 Infinity HPLC (Agilent Technologies) coupled to a 6560 IMS-QTOF-MS (Agilent Technologies) was used. For chromatographic separation, solvent A consisted of 60% 18 MΩ-water and 40% acetonitrile with 10 mM ammonium acetate and 1 mM acetic acid, and solvent B was 90% isopropanol, and 10% acetonitrile with 10 mM ammonium acetate and 1 mM acetic acid. The used HPLC gradient started with 60% solvent A and 40% solvent B for 8 min followed by 11 min of 30% A and 70% B and a final step to 10% A and 90% B for 9 min. The flow was 0.7 mL/min, and the injection volume was set to 3 µL. The mass spectrometer was equipped with a Dual Agilent Jet Stream Electrospray Ionization (Dual AJS-ESI, Agilent, Waldbronn, Germany) source, which was operated in positive mode. The scan range was 100 to 1200 m/z. Ion mobility measurement was completed by using N_2_ as drift gas and 4-bit multiplexing with a trap fill time of 3900 µs and a trap release time of 250 µs.

### 2.4. Data Processing and Statistical Analysis

The generated raw data was processed by PNNL PreProcessor 3.0 (Version 24 November 2020.) for demultiplexing and chromatographic smoothing. IM-MS Browser (Version 10.0), Mass Profiler (Version 10.0), and PCDL Manager (Version 8.00), all from Agilent Technologies (Waldbronn, Germany), with an in-house library, were used for feature extraction and feature annotation. Statistical analysis was carried out employing the web-based platform of MetaboAnalyst (McGill University, Montreal, QC, Canada). The visualization of the boxplots was completed with OriginPro 2021 (Northampton, MA, USA).

## 3. Results

### 3.1. Study Subjects

In total, 52 patients with SSc and 48 controls without SSc were recruited. SSc patients had a mean age of 60 years, and 44 patients were female. Seventeen patients were positive for anti-Scl70-antibodies (anti-topoisomerase I-antibodies), eighteen patients showed positivity towards anti-centromere-antibodies (ACA), thirteen patients were only ANA-positive, and four patients had anti-SSA (alone or combined with anti-Rnp-Sm, anti-Pm-scl75, or anti-Pm-Scl100). Eleven patients had diffuse cutaneous SSc (dcSSc), 39 patients had limited cutaneous SSc (lcSSc), and two patients had no skin sclerosis (both were female patients with Very Early diagnosis of Systemic Sclerosis, VEDOSS). The characteristics of the patients with systemic sclerosis (SSc) and control group (without SSc) are summarized in Table 1.

### 3.2. Plasmalogens and Sphingolipids from Plasma Are Increased in Patients with SSc

Plasma samples have been evaluated for 263 targets. Within the volcano plot (Supplementary Figure S1), it can be observed that in SSc, PC ae, and SM species were up-regulated. Furthermore, within the top 25 features based on the T-tests result in the heatmap, a tendency of clustering due to these lipids was observed (Supplementary Figure S2). This was more visible within a heatmap only showing groups (Supplementary Figure S3). The statistical results for the top features based on *p*-value resulting from the *T*-tests and fold changes are listed in the Supplementary Material (Supplementary Tables S1 and S2).

**Figure 1:**
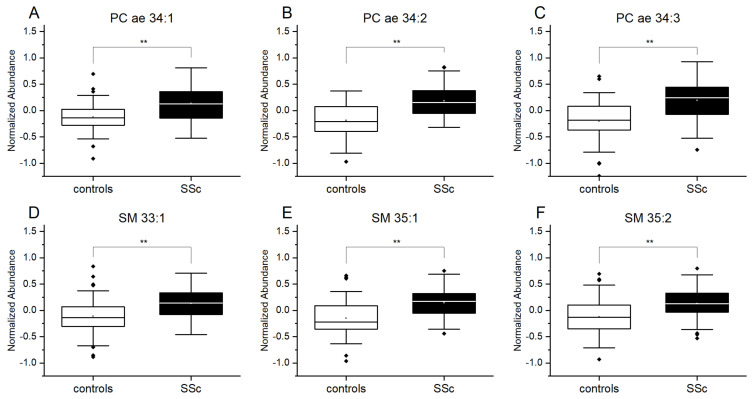
Increased plasma levels of plasmalogens and sphingolipids in patients with SSc. Results of lipidomics measurements for (**A**) phosphatidylcholine PC ae 34:1, (**B**) PC ae 34:2, (**C**) PC ae 34:3 and for (**D**) sphingomyelin SM 33:1, (**E**) SM 35:1 and (**F**) SM 35:2. Controls (*n* = 48), SSc patients (*n* = 52). Data are expressed as normalized abundance. Resulting *p*-values from group comparisons were determined using a two-tailed Student’s *t*-test, and *p* < 0.05 was considered significant (** *p* < 0.01).

The data showed that there was a significant increase in the levels of PC ae (PC ae 34:1, 34:2, 34:3) and SM (SM 33:1, 35:1, 35:2) in patients with SSc as compared to the controls subject without SSc (Figure 1).

### 3.3. Differences in Plasmalogens and Sphingolipids from Plasma in Patients with lcSSc and dcSSc

**Figure 2:**
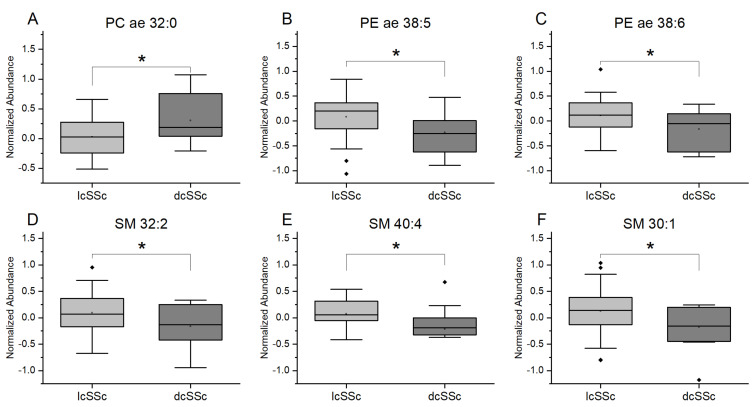
Differences in plasma levels of plasmalogens and sphingomyelins between dcSSC and lcSSc patients. Results of lipidomics measurements for (**A**) phosphatidylcholine PC ae 32:0, (**B**) phosphatidylethanolamine PE ae 38:5, (**C**) PE ae 38:6 and for (**D**) sphingomyelin SM 32:2, (**E**) SM 40:4 and (**F**) SM 30:1. Limited cutaneous systemic sclerosis, lcSSc (*n* = 39), diffuse cutaneous systemic sclerosis, dcSSc (*n* = 11). Data are expressed as normalized abundance. Resulting *p*-values from group comparisons were determined by a two-tailed Student’s *t*-test, and *p* < 0.05 was considered significant (* *p* < 0.05).

We further compared plasma lipid levels of patients with dcSSc and patients with lcSSc. Data showed a statistically significant increase in PC ae 32:0 species and a decrease in PE ae 38:5 and PE ae 38:6 in the dcSSc group as compared with the lcSSc patients. Moreover, SM (SM 30:1, SM 32:2, and SM 40:4) were significantly decreased in dcSSc patients (Figure 2).

### 3.4. Plasmalogens and Sphingolipids from Plasma Are Associated with Clinical Symptoms in SSc Patients

**Figure 3:**
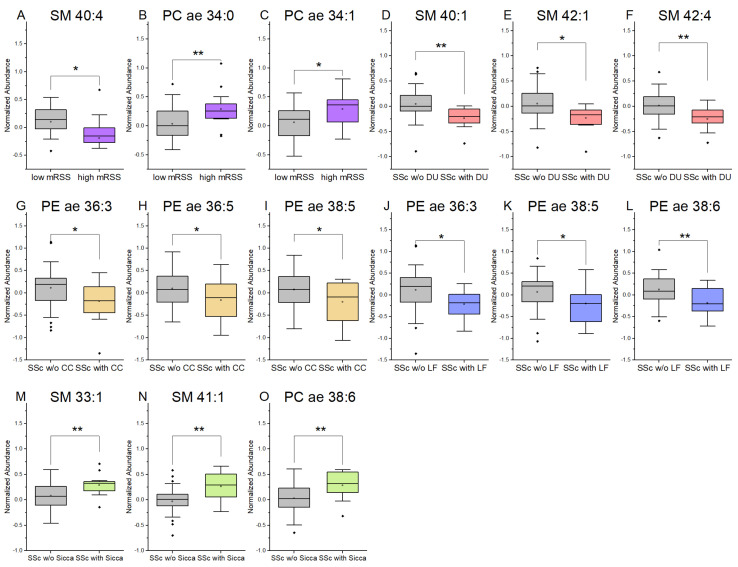
Association of plasmalogens and sphingomyelins from plasma with clinical manifestations of SSc. Results of lipidomics measurements for (**A**) sphingomyelin, SM 40:4, (**B**) phosphatidylcholine PC ae 34:0, (**C**) PC ae 34.1, (**D**) SM 40:1, (**E**) SM 42:1, (**F**) SM 42:4 (**G**) phosphatidylethanolamine PE ae 36:3, (**H**) PE ae 36:5 and (**I**) PE ae 38:5, (**J**) PE ae 36:3, (**K**) PE ae 38:5, (**L**) PE ae 38:6, (**M**) SM 33:1, (**N**) SM 41:1, and (**O**) PC ae 38:6. Modified Rodnan skin score, mRSS (*n* = 15), digital ulcers, DU (*n* = 10), calcinosis cutis, CC (*n* = 15), lung fibrosis, LF (*n* = 14), sicca (*n* = 14). Data are expressed as normalized abundance. Resulting *p*-values from group comparisons were determined by a two-tailed Student’s *t*-test, and *p* < 0.05 was considered significant (* *p* < 0.05, ** *p* < 0.01).

To further examine whether changes in plasma lipid levels were linked to symptoms of SSc, we analyzed plasmalogens and sphingolipids in subgroups of SSc patients. The data showed that in SSc patients with high mRSS (>14), SM (SM 40.4) were significantly decreased, and plasmalogens (PC ae 34:1, PC ae 34.0) were significantly increased as compared to patients with low mRSS (Figure 3A–C). In SSc patients with digital ulcers (DU), significantly decreased plasma levels of SM (SM 40:1, SM 42:1, and SM 42:4) were observed, as compared to SSc patients without DU (Figure 3D–F). Calcinosis cutis in SSc patients was significantly associated with decreased levels of plasmalogens in plasma (Figure 3G–I). SSc patients with lung fibrosis (LF) had significantly lower PE plasmalogen levels in plasma (PE ae 36:3, PE ae 38:5, and PE ae 38:6) as compared to SSc patients without LF (Figure 3J–L). Significantly increased levels of PC plasmalogens were associated with sicca symptoms in SSs patients (Figure 3M–O).

## 4. Discussion

In the present study, we analyzed the lipidomic profile of plasma from SSc patients from a single cohort (Upper Austria) compared to controls. The study’s major findings were significantly altered phospholipids such as plasmalogens and sphingomyelins in plasma from SSc patients versus controls and association of changed phospholipid metabolism with different symptoms of SSc.

We detected significantly increased plasma levels of PC ae in the plasma of SSc patients (PC ae 34:1, PC ae 34:2 and PC ae 34:3) versus controls, and in the plasma of dcSSc patients (PC ae 32:0) versus lcSSc patients. To our knowledge, no data are published on the role of PC ae in SSc. One study showed that linoleic acid (18:2) proportion in serum PC was lower in SSc patients than in control subjects [29]. The previous study reported significantly higher serum levels of phospholipids other than PC, such as arachidonoyl (20:4)—LPA, and S1P, in SSc versus controls [12]. The role of LPA and S1P in SSc as relevant for platelet-activation, antigen-processing in dendritic cells, or early fibrogenesis during SSc was suggested [12,30].

Several studies described the possible role of PC in different diseases, others from SSc. The lipidomic study showed a significant decrease in some species of PC in patients with SLE [5]. Oxidative stress led to the lower levels of plasmalogens in sera from patients with SLE, and oxidation-related lipids have been shown as promising novel biomarkers for SLE diagnosis [31]. Analyses of lipidomics in dermatomyositis and polymyositis revealed the identification of three PC (PC 38:4, PC 36:4, and PC 40:8) that were significantly decreased in patients compared to controls [10]. In mucus from patients with ulcerative colitis, a low PC level was shown, while oral intake of PC improved intestinal function in the animal model [32,33]. Lipidomic profiling showed the down-regulated metabolism of PC in psoriasis patients [34]. PC levels from the serum of RA patients were found to be correlated to the disease duration of RA. [35]. The role of PC ae in SSc patients has to be further explored.

We found significantly decreased levels of PE ae in plasma from dcSSc patients (PE ae 38:5 and PE ae 38:6) as compared with lcSSc patients. The role of PE species in SSc is unknown. Recent data showed that some SSc patients have anti-PE-IgM antibodies, and these were associated with hypocomplementemia [36]. Moreover, PE were shown to induce antifibrotic mechanisms in lung fibrosis [37]. Our data showed that lung fibrosis in SSc patients was associated with decreased plasma levels of PE ae (PE ae 36:3, PE ae 38:5, PE ae 38:6). Thus, certain PE species could be related to the severity of fibrosis during SSc.

Our data showed significantly higher SM levels in plasma from SSc patients (SM 33:1, SM 35:1, and SM 35:2) versus controls. Previous data demonstrated that sphingolipids have an important role in the healing of wounds and are strongly involved in TGF-β signaling and the regulation of fibrosis in the lungs and skin [38]. Sphingolipids such as S1P and ceramide were shown to regulate inflammation and stimulate renal fibrosis [39]. Moreover, in patients with psoriasis, an increase in circulating S1P levels is described. [40] Our data further showed significantly decreased sphingomyelins in the plasma of patients with dcSSc (SM 30:1, SM 32:2 and SM 40:4) versus lcSSc, in plasma of SSc patients with more intensive skin sclerosis with mRSS > 14 (SM 40.4), and in SSc patients with DU (SM 40:1, SM 42:1, and SM 42:4). Thus, it seems that certain species of sphingomyelins can influence skin sclerosis and chronic wounds in patients with SSc. Further studies are needed to elucidate the role of sphingomyelins in SSc.

Calcinosis cutis (CC) in SSc patients often leads to high impairment of the quality of life in SSc patients due to complications such as chronic wounds, pain, superinfections, sepsis, and hospitalization. Efficient therapy for CC is lacking, and very little is known about the pathophysiology of the CC in SSc. Our data showed that CC in SSc patients was associated with decreased plasma levels of PE. A previous publication reported that PE plays an essential role in vascular calcification as a significant component of the autophagosome membrane [41,42]. Autophagosomes contain calcium, phosphate, and calcified hydroxyapatite, which can be released during calcification. Lipids such as PE were described to function as nucleation centers for the formation of calcinosis [43,44]. Thus, PE might also play an essential role for CC in SSc patients.

Sicca symptoms can decrease the quality of life in SSc patients due to pain and complications on the eyes, mouth, or nose. Therapeutic options are limited. Our data showed that sicca symptoms in SSc patients were significantly associated with decreased PC ae and SM in plasma. This is in line with previously published data showing low levels of PE and SM in the ocular fluid, which was associated with ocular surface abnormalities in sicca patients [45]. Recently, a study was published describing the positive effects of oral uptake of choline alfoscerate (precursor of acetylcholine) in patients with sicca symptoms, and PC was shown to stimulate the synthesis of acetylcholine [46,47]. Thus, PC, PE, and SM play an important role in pathophysiology, and PC possibly also in the therapy of sicca symptoms, which could be true also for SSc patients with sicca problems and should be further evaluated.

In addition to the role of lipid plasmalogens in inflammation and fibrosis, one further aspect could be involved in SSc. Lipid plasmalogens also function as antioxidants and can influence cell differentiation. The exact role of plasmalogens has still to be explored in detail, but data are suggesting that lipid plasmalogens and their metabolites may play an essential role in the development of cancer diseases, especially adenocarcinoma [48]. Previous studies showed that lipid plasmalogens are potential biomarkers for the prognosis of adenocarcinoma such as breast, lung, colorectal, gastric, ovarian, or prostate carcinoma [49,50,51,52,53]. Moreover, synthetic plasmalogens have a promising role in the chemotherapy of tumor diseases [54,55]. Patients with SSc have a higher risk for adenocarcinoma as compared to healthy individuals [56]. This is primarily due to chronic inflammation during SSc, and for lung cancer, due to reflux and fibrosis. Due to improved medical care for SSc patients and more prolonged survival, cancer disease in SSc patients became one of the significant causes of death [57]. We hypothesized that lipid plasmalogens in SSc patients might have an important role for carcinogenesis in SSc patients and may in the future be useful as a prognostic biomarker or maybe even as a therapeutic agent in patients with SSc-associated malignancies. Further studies are needed to assess the role of plasmalogens in carcinogenesis in SSc patients.

In conclusion, previous studies have suggested that lipids may play an important role in autoimmune, inflammatory, and cancer diseases. The lipidomic approach led to the discovery of lipids in several autoimmune diseases such as SLE, RA, multiple sclerosis, which can be potentially used as biomarkers of the disease.

Our present lipidomic study described, for the first time, significant changes in the level of phospholipids such as plasmalogens and sphingomyelins from plasma of SSc patients and the association of changed phospholipid metabolism with different clinical manifestations of SSc.

The limitations of our study were a single small cohort and single time-point examination. Future studies should explore longitudinal lipidomic profiles during the natural course of SSc and compare lipidomic data before and after treatment of SSc disease in a large multi-center SSc cohort.

Lipidomic studies might contribute to the identification of new lipids, which could be relevant in the pathophysiology of SSc. This could lead to the detection of new biomarkers for prognosis and treatment monitoring of SSc. Moreover, targeting lipids or their receptors based on lipidomics data could lead to the development of new therapies of SSc.

## Figures and Tables

**Table 1:** Demographic and clinical characteristics of patients with systemic sclerosis (SSc) and controls.

Characteristics	SSc (*n* = 52)	Controls (*n* = 48)
Female/Male	44/8	37/11
Age (range)	60 ± 12	51 ± 16
Anti-Scl70/ACA, *n* (%)	17 (32.7%)/18 (34.6%)	-
ANA only/other Abs, *n* (%)	13 (25%)/4 (7.7%)	
dcSSc/lcSSc, *n* (%)	11 (21.2%)/39 (75%)	-
Lung fibrosis (LF), *n* (%)	14 (26.7%)	
Digital ulcers (DU), *n* (%)	10 (19.2%)	-
Calcinosis cutis (CC), *n* (%)	15 (28.8%)	-
Sicca symptoms, *n* (%)	14 (26.9%)	-
Raynaud symptoms, *n* (%)	44 (84.7%)	-
Pulmonal arterial hypertension, *n* (%)	20 (38.5%)	-
Mycophenolate mofetil, *n* (%)	5 (9.6%)	-
Methotrexate, *n* (%)	14 (26.9%)	-
Bosentan, *n* (%)	8 (15.4%)	-
Ambrisentan/Macitentan, *n* (%)	2 (3.8%)/10 (19.2%)	-

## Data Availability

The data presented in this study are available on request from the authors M.G.-S. and T.B.

## Supplementary Materials

The following are available online: https://www.mdpi.com/article/10.3390/diagnostics11112116/s1, Figure S1: Volcano plot indicates increased plasma levels of plasmalogens and sphingolipids in patients with SSc; Figure S2: Heatmap combined with clustering using a Euclidean distance measure and ward algorithm; Figure S3: Heatmap combined with clustering using a Euclidean distance measure and ward algorithm showing only group averages; Table S1: Significant results of *t*-tests (*p*-value < 0.05); Table S2: Significant results of fold changes (>2).
