# Supplementary material for: Changes in Plasma Phospholipid Metabolism Are Associated with Clinical Manifestations of Systemic Sclerosis
Source: Diagnostics (Basel). 2021 Nov 15;11(11):2116. doi: 10.3390/diagnostics11112116 (PMC8625116; doi:10.3390/diagnostics11112116)
Supplement: Supplementary file 1 [file diagnostics-11-02116-s001.zip › diagnostics-1396956-supplementary.pdf]

Supplementary Information – Data Analysis

Detailed information regarding performed statistical analysis and results.

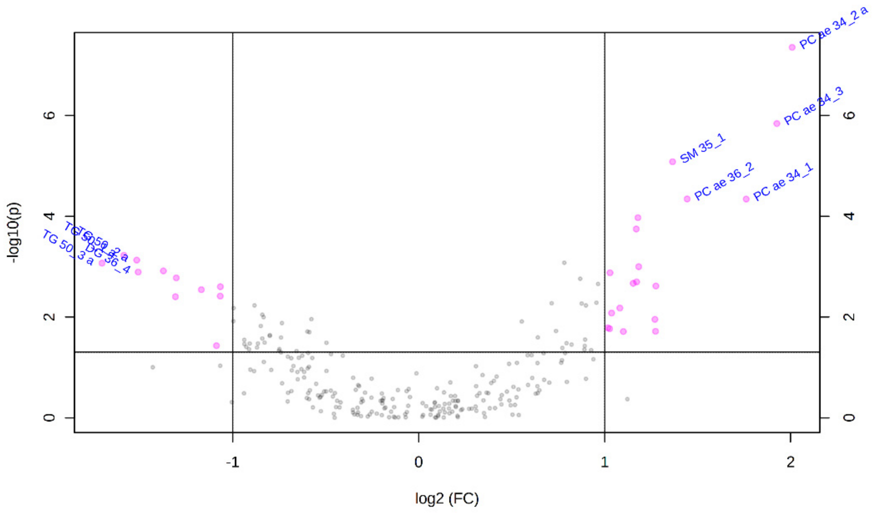

Supplementary Figure S1. Volcano plot indicates increased plasma levels of plasmalogens and sphingolipids in patients with SSc.

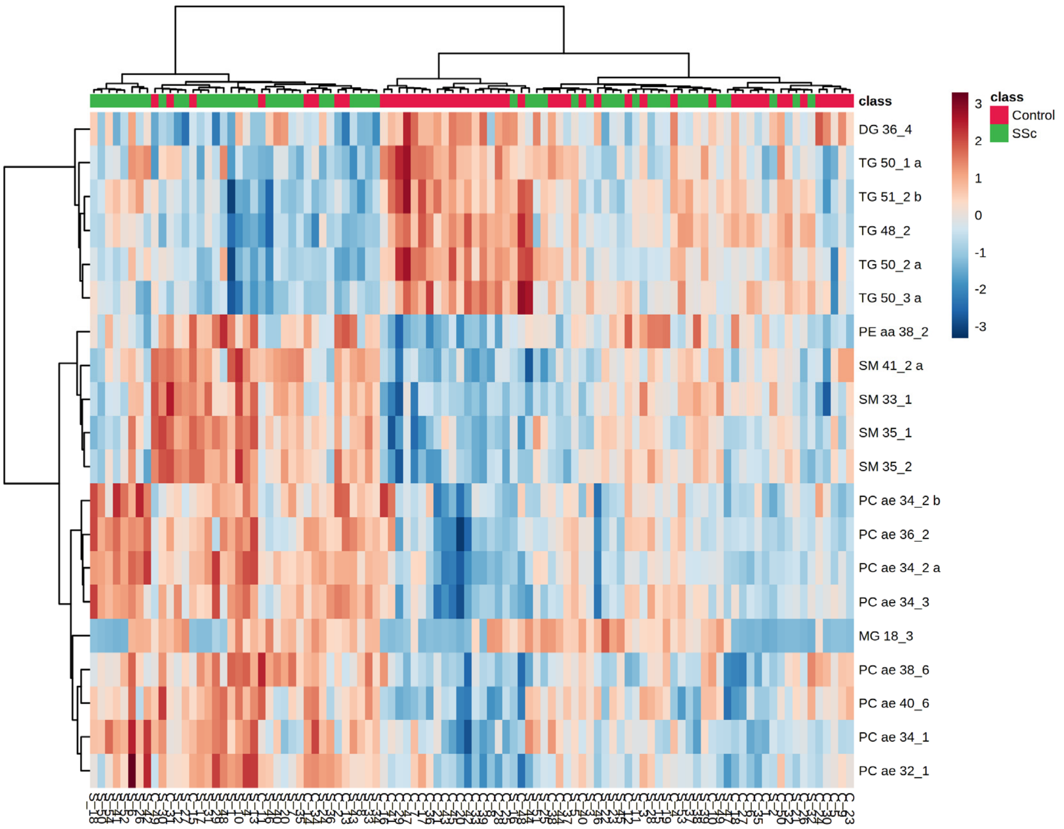

Supplementary Figure S2. Heatmap combined with clustering using a euclidean distance measure and ward algorithm

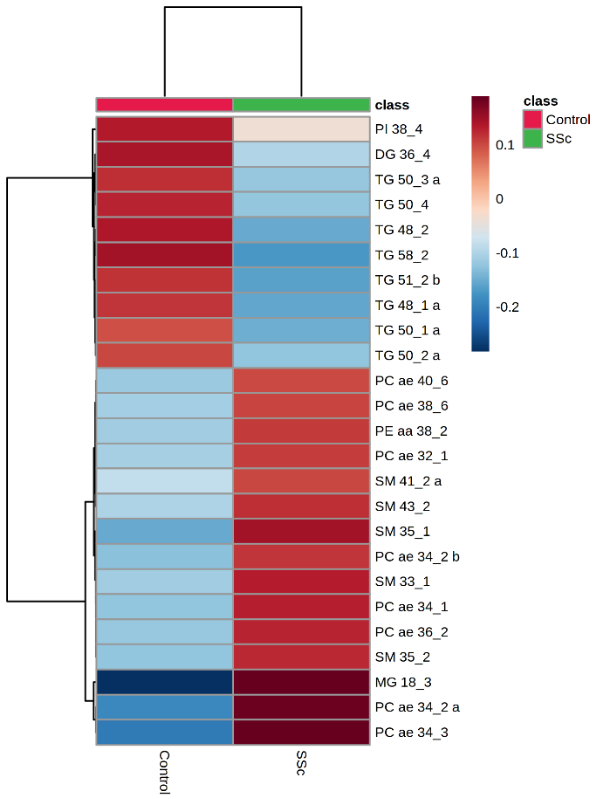

Supplementary Figure S3. Heatmap combined with clustering using a euclidean distance measure and ward algorithm showing only group averages.

Supplementary Table S1. Significant results of t-tests (p-value <0.05).

Lipid	t.stat	p.value	$-log^{10}(p)$	FDR
PC ae 34_2 a	-5.9358	4.43E-08	7.3534	1.17E-05
PC ae 34_3	-5.1334	1.44E-06	5.8407	0.00018979
SM 35_1	-4.707	8.27E-06	5.0825	0.00072499
PC ae 36_2	-4.2697	4.53E-05	4.3439	0.0023965
PC ae 34_1	-4.2681	4.56E-05	4.3414	0.0023965
SM 33_1	-4.0393	0.00010661	3.9722	0.004673
SM 35_2	-3.8958	0.00017897	3.7472	0.0067242
TG 50_1 a	3.5495	0.00059466	3.2257	0.019549
TG 50_2 a	3.4837	0.00074095	3.1302	0.020375
PE aa 38_2	-3.4472	0.00083585	3.0779	0.020375
TG 50_3 a	3.4414	0.00085217	3.0695	0.020375
PC ae 38_6	-3.3922	0.0010013	2.9994	0.021945
TG 51_2 b	3.3328	0.001214	2.9158	0.023196
DG 36_4	3.3162	0.0012807	2.8925	0.023196
PC ae 40_6	-3.3061	0.001323	2.8784	0.023196
TG 48_2	3.2326	0.0016714	2.7769	0.02688
MG 18_3	-3.2204	0.0017375	2.7601	0.02688
PC ae 32_1	-3.1743	0.0020073	2.6974	0.029006
SM 41_2 a	-3.1536	0.0021411	2.6694	0.029006
PC ae 34_2 b	-3.144	0.0022057	2.6564	0.029006
SM 43_2	-3.1141	0.0024192	2.6163	0.029887
PI 38_4	3.1034	0.0025001	2.602	0.029887
TG 58_2	3.0593	0.002861	2.5435	0.032715
TG 50_4	2.9617	0.0038382	2.4159	0.04157
TG 48_1 a	2.952	0.0039515	2.4032	0.04157
PE aa 36_0	-2.8591	0.0051906	2.2848	0.050571
PC ae 36_6	-2.85	0.0053297	2.2733	0.050571
PE aa 38_1	-2.8465	0.005384	2.2689	0.050571
PE aa 34_0	-2.8159	0.0058817	2.2305	0.051575
TG 48_3	2.8158	0.0058831	2.2304	0.051575
PC ae 34_0	-2.7743	0.0066257	2.1788	0.054611
TG 46_1	2.7732	0.0066447	2.1775	0.054611
SM 34_1	-2.6928	0.0083352	2.0791	0.066429
TG 46_2	2.6661	0.0089761	2.0469	0.069433
PC aa 38_3	2.6246	0.010065	1.9972	0.075629
TG 46_3	2.5917	0.011009	1.9583	0.079133
PC ae 32_1 b	-2.5876	0.011133	1.9534	0.079133
TG 54_2	2.5583	0.012051	1.919	0.082616
SM 42_3	-2.5521	0.012251	1.9118	0.082616
TG 45_2	2.5242	0.013203	1.8793	0.086812
DG 38_4 b	2.4744	0.015066	1.822	0.096643
PC ae 38_6 b	-2.4432	0.016347	1.7866	0.10236
SM 31_1	-2.4271	0.017048	1.7683	0.10427
LPE 20_0	-2.3863	0.01894	1.7226	0.10866
PC ae 32_0	-2.381	0.019197	1.7168	0.10866

PE aa 42_5	-2.3767	0.019411	1.712	0.10866
PC aa 36_4 a	2.3766	0.019418	1.7118	0.10866
TG 58_4	2.3666	0.019916	1.7008	0.10912
DG 38_9	2.3127	0.022828	1.6415	0.12253
TG 44_1	2.2973	0.023731	1.6247	0.12483
Octadecenoylcarnitine	-2.2704	0.02537	1.5957	0.12901
TG 52_5	2.2683	0.025507	1.5933	0.12901
TG 47_2	2.2503	0.026668	1.574	0.13233
PC ae 36_5 a	-2.2303	0.028011	1.5527	0.13581
TG 49_1 b	2.2246	0.028402	1.5467	0.13581
TG 50_5	2.1728	0.032203	1.4921	0.14709
PI 36_1	2.1696	0.032456	1.4887	0.14709
PC ae 36_5 b	-2.1616	0.033087	1.4803	0.14709
PC aa 40_4	2.1563	0.03351	1.4748	0.14709
PC aa 36_3 a	2.1557	0.033557	1.4742	0.14709
TG 58_5	2.1453	0.034396	1.4635	0.1483
PE aa 42_6	-2.1363	0.035143	1.4542	0.14908
DG 34_1	2.1144	0.037015	1.4316	0.15367
PE aa 40_5	-2.1101	0.037395	1.4272	0.15367
DG 36_1	2.0909	0.039121	1.4076	0.15829
TG 45_1	2.0785	0.040275	1.395	0.16049
SM 38_2	-2.0702	0.041059	1.3866	0.16117
TG 48_4	2.0475	0.043285	1.3637	0.16616
DG 40_4	2.0436	0.043681	1.3597	0.16616
PE ae 40_6 b	-2.0382	0.044225	1.3543	0.16616
PC ae 36_3 a	-2.0273	0.045344	1.3435	0.16796
DG 38_4	2.0156	0.046577	1.3318	0.17014
GlcCer 38_1 OH	2.0085	0.047337	1.3248	0.17054

Supplementary Table S2. Significant results of fold changes (>2).

Lipid	Fold Change	log2(FC)
PC ae 34_2 a	4.0237	2.0085
PC ae 34_3	3.8004	1.9262
PC ae 34_1	3.3898	1.7612
TG 50_3 a	0.30703	-1.7035
TG 50_1 a	0.33307	-1.5861
TG 50_2 a	0.34946	-1.5168
DG 36_4	0.35137	-1.5089
PC ae 36_2	2.7202	1.4437
PE aa 36_2 b	0.37106	-1.4303
TG 51_2 b	0.386	-1.3733
SM 35_1	2.5761	1.3652
TG 48_1 a	0.40371	-1.3086
TG 48_2	0.40497	-1.3041
SM 43_2	2.4209	1.2755
PC ae 32_0	2.4177	1.2736

PC ae 32_1 b	2.4121	1.2703
PC ae 38_6	2.2706	1.1831
SM 33_1	2.2641	1.1789
PC ae 32_1	2.2529	1.1718
SM 35_2	2.2501	1.17
TG 58_2	0.44467	-1.1692
SM 41_2 a	2.2247	1.1536
SM 44_6	2.177	1.1223
PE aa 42_5	2.1442	1.1005
DG 34_1	0.47043	-1.0879
PC ae 34_0	2.1166	1.0817
PE aa 34_2	0.47708	-1.0677
TG 50_4	0.4771	-1.0676
PI 38_4	0.47734	-1.0669
SM 34_1	2.0523	1.0372
PC ae 40_6	2.0402	1.0287
SM 31_1	2.0378	1.027
PC ae 38_6 b	2.024	1.0172
TG 55_2	0.4981	-1.0055
